# Revision for dislocation and all-causes following primary total hip replacement using 36-mm versus 32-mm femoral heads on polyethylene liners: a systematic review and meta-analysis

**DOI:** 10.1007/s00402-025-06151-w

**Published:** 2025-12-15

**Authors:** Muhamed M. Farhan-Alanie, Mina Abdul-Hussein, Daniel Gallacher, Prakrit R. Kumar, Rajpreet Sahemey, Peter D. H. Wall, Michael Blankstein

**Affiliations:** 1 University of Warwick, Coventry, UK; 2 Imperial College London, London, UK; 3 University of Birmingham, Birmingham, UK; 4 University Hospitals Birmingham NHS Foundation Trust, Birmingham, UK; 5 University of Vermont, Burlington, USA

**Keywords:** THR, Head size, Revision, Dislocation, Loosening

## Abstract

**Introduction:**

The use of a 36-mm femoral head in primary total hip replacement (THR) increases the jump distance and offers a wider impingement-free range of motion, theoretically reducing the risk of post-operative dislocation. However, concerns exist regarding its potentially greater impact on polyethylene wear and associated risks of liner fracture, aseptic loosening, and head-neck taper corrosion, compared to 32-mm femoral heads. This meta-analysis aims to compare the risk of revision for dislocation and all-causes when using 36-mm versus 32-mm metal or ceramic femoral heads with polyethylene liners for primary THR.

**Methods:**

Medline, Embase, Web of Science, and the Cochrane Library were searched for relevant studies, while annual reports of arthroplasty registries were searched for relevant data. Random effects meta-analysis was performed. Sensitivity analysis was conducted, limiting to THR performed for osteoarthritis and using cross-linked polyethylene liners, or statistically adjusting for this factor. The study was registered in the International Prospective Register of Systematic Reviews (PROSPERO ID CRD42024557895).

**Results:**

Four observational studies and two registry reports were identified. Median follow-up ranged from 2.1 to 4.7 years (inter-quartile range values spanned from 0.9 to 7.7 years). The main analyses demonstrated that 36-mm heads were associated with a marginal reduction in the risk of revision for dislocation (HR 0.85, 95%CI 0.72–1.01, *p* = 0.058; *n* = 241,136) without an increased risk of all-cause revision (HR 1.06, 95%CI 0.95–1.18, *p* = 0.287; *n* = 942,617). Similar results were observed in the sensitivity analyses.

**Discussion:**

At early to midterm follow-up, the use of 36-mm heads were not associated with an increased risk of revision for all-causes, compared to 32-mm heads. However, they may offer a protective reduction against revision for dislocation, as a marginal statistically significant reduction was observed, indicating a possible benefit. Further studies investigating these outcomes at longer-term follow up are needed to understand whether these revision risk profiles are maintained.

**Supplementary Information:**

The online version contains supplementary material available at 10.1007/s00402-025-06151-w.

## Introduction

The incidence of dislocation following primary total hip replacement (THR) ranges from 1.7% to 3.5% [1–3] and it is associated with both lower patient reported health- and hip-related quality of life [4]. Treatment for prosthetic hip dislocation typically involves a closed reduction or revision procedure, both of which are associated with substantial healthcare costs [5, 6]. Furthermore, patients undergoing revision surgery for dislocation have relatively inferior Oxford Hip Scores compared to those who have never experienced a dislocation [7]. The use of larger prosthetic femoral head sizes in THR theoretically provide greater stability by increasing the jumping distance and allowing a wider impingement-free range of motion due to the increased head-neck ratio [8, 9]. Although larger femoral head sizes may reduce the risk of dislocation, there are concerns they may increase the risk of revision for other causes including aseptic loosening. This is believed to be due to the relatively increased polyethylene wear and their associated particles migrating into the joint space, stimulating a foreign body response that results in periprosthetic osteolysis [10–13]. Moreover, larger femoral heads have also been shown to be associated with increased frictional torque that is believed to result in mechanically assisted crevice corrosion that can also cause osteolysis and implant loosening as well as pain, and other symptoms experienced by patients [14–20].

Current practice regarding femoral head size varies both nationally and internationally. According to the latest published annual reports from the national joint registries of England and Germany, primary THR are performed using 36-mm and 32-mm femoral head sizes in approximately equal proportions [21, 22]. Conversely, the most common femoral head size in Sweden has been 32-mm since 2010, while the proportion of 36-mm femoral heads used has remained consistent at approximately 20% since 2013 [23]. Similarly, New Zealand’s latest annual report shows that 32-mm femoral heads were the most commonly used during the years 2010 to 2022 [24]. In contrast, the most common femoral head size used in the United States and Denmark is 36 mm, a trend that has persisted since 2012 [25, 26].

The introduction of highly cross-linked polyethylene (HXLPE) liners have markedly reduced wear rates compared to conventional polyethylene liners [27]. However, it remains unclear whether HXLPE offers comparable resistance to wear when using 36-mm versus 32-mm femoral heads. Clinical studies investigating this topic using radiographic and radiostereometric evaluations have found conflicting results [28–30]. Furthermore, as some of these studies have a maximum follow-up of 10 years post-operatively, the long-term performance and comparative risk of revision associated with these femoral head sizes remain uncertain. These studies also do not provide insights regarding the risk of dislocation. Despite these uncertainties between these two femoral sizes, the perceived benefits of the 36-mm heads have led surgeons to increasingly adopt their use [15, 31]. By pooling the available data within the literature and comparing the difference in the rates of revision surgery between these two sizes of femoral heads, better evidence may be obtained about their clinical effectiveness in THR. The aim of this systematic review and meta-analysis is to compare the risk of revision for dislocation and all-causes when using 36-mm versus 32-mm metal or ceramic femoral heads with a polyethylene liner for primary total hip replacement.

## Methods

### Data sources and search strategy

The study adhered to the PRISMA (Preferred Reporting Items for Systematic Review and Meta-Analysis) and MOOSE (Meta-Analysis of Observational Studies in Epidemiology) guidelines [32, 33]. Medline, Embase, Web of Science, and Cochrane Library were searched from their inception up to 6th September 2024. The search strategy is reported in table S1. Two authors (MFA, MH) independently assessed articles for inclusion. Articles were initially screened by title and abstract, followed by full text review of selected articles. Bibliographies of identified relevant articles were manually scanned to identify any missed relevant articles. Additionally, the published annual reports of the Members of the International Society of Arthroplasty Registries were manually searched for relevant data [34]. The study was registered in PROSPERO (International Prospective Register of Systematic Reviews; CRD42024557895).

### Eligibility criteria

Randomised controlled trials (RCTs) and comparative observational studies were eligible for inclusion. Eligible studies included those reporting on revision for dislocation, aseptic causes, or all-causes following the use of 32-mm versus 36-mm metal or ceramic femoral head with a polyethylene liner in primary THR for any indication. The outcomes of interest were revision for all-causes and revision for dislocation. We included studies that reported on revisions for aseptic causes in our analysis of all-cause revisions, as femoral head size is not associated with prosthetic joint infection, thus these events would be expected to occur in similar proportions between groups [35]. Studies that focussed on patients with specific comorbidities undergoing THR (e.g. lumbar spinal fusion) or analysed patients undergoing revision procedures were excluded. Studies that compared multiple head sizes that were categorised within groups or included bearings other than Metal-on-Polyethylene (MoP) and Ceramic-on-Polyethylene (CoP) were excluded. Where publications investigated the same patient cohort, the most comprehensive study with longest follow-up was included. Studies in English or with an accessible translation were included. Animal studies and in vitro studies were excluded. Disagreements regarding study inclusions and marginal studies for eligibility were discussed with the senior author (MB) prior to inclusion or exclusion.

### Data extraction and quality assessment

Data was extracted using a standardised form. Methodological quality was assessed on the Newcastle-Ottawa Scale [36]. This assesses three domains (selection, comparability, and outcome) across eight items with each scoring one point except for comparability which can be adapted to the specific topic to score up to two points. The two risk factors selected a priori to be most pragmatic and relevant to control for confounding between the comparison groups were patient’s age and the use of cross-linked polyethylene. Studies scoring less than five points were judged as high risk of bias and excluded, five to seven were considered medium risk, and eight or nine points were deemed low risk.

### Data synthesis and statistical analysis

A multilevel meta-analysis was conducted using the ‘metafor’ package in R (v4.1.2, R Core Team, 2021. Vienna, Austria) using a single effect estimate from each study [37]. Where a study reported multiple relevant effect estimates, a within study meta-analysis was performed to obtain a single study estimate of effect. An inverse variance method was used and random-effects models were applied due to interstudy heterogeneity. Adjusted hazard ratios (HR) from Cox proportional hazards regression models were extracted from the included studies. Where hazard ratios were not reported, such as in published annual reports of national arthroplasty registries, Kaplan-Meier survival curves were digitised and data coordinates extracted using WebPlotDigitizer 4.7 (Automeris, 2024), with hazard ratios estimated from a Cox proportional hazards model fitted to the recreated pseudo-data using the ‘ipdfc’ command within Stata (version 18.0, StataCorp LLC, College Station, Texas, USA, 1985–2023) [38]. A sensitivity analysis was performed limiting to patients with osteoarthritis who underwent THR using cross-linked polyethylene liners or statistically adjusting for this factor. Higgins I^2^ test was performed to provide an estimate of the extent of statistical heterogeneity and values were interpreted in accordance with the Cochrane Handbook [39]. 95% confidence intervals (CI) were calculated for each study with statistical significance set at *p* < 0.05. Summary estimates of the overall effect are provided as a forest plot. Narrative discussion has been provided where statistical analysis was not possible.

## Results

### Study identification and selection

**Fig. 1:**
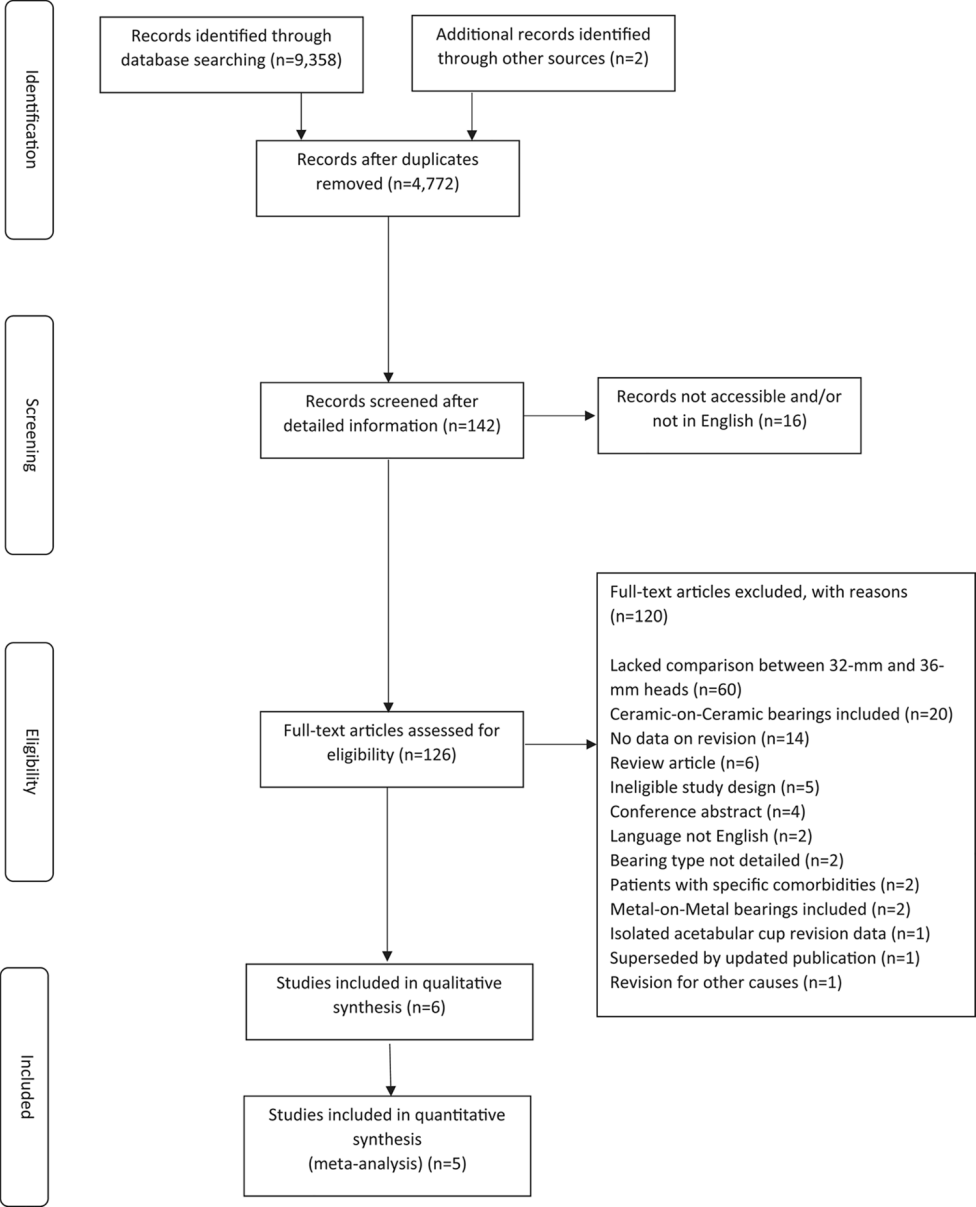
PRISMA flow diagram

The literature search identified 4,772 articles. Study selection is illustrated in Fig. 1. Six publications met the inclusion criteria including four comparative observational studies of arthroplasty registry datasets [40, 43]. Data from annual reports of two arthroplasty registries were also suitable for inclusion [21, 44]. Included publications are detailed in Table 1.

**Table 1:** Description of included studies

Publication Year	First Author	Study Design	Study Time Period	Country	Study population	Age (Years) (32 mm/36 mm)	Sex (Male: Female) (%) (32 mm/36 mm)	Mean BMI (32 mm/36 mm)	Majority ASA (32 mm/36 mm)	Total Procedures (32 mm/36mmm)	CoP Procedures (32 mm/36 mm)	MoP Procedures (32 mm/36 mm	Outcome(s) Measured	Follow-Up Duration (Years)	Covariate Adjustments
2022	Hoskins	Cohort Study	September 1999 – December 2019	Australia	Osteoarthritis	Median 70 (IQR# 63–77) for 32 mm and median 71 (IQR 64–77) for 36 mm	38.6:61.4/58.2:41.8	Mode 25.00–29.99 kg/m^2^/mode 25.00–29.99 kg/m^2^	2.31/2.32	150,969 (84,391/66,578)	Not reported	Not reported	Revision for all aseptic causes and revision for dislocation	Median 4.7 (IQR 2.4–7.7) for 32 mm and median 3.9 (IQR 1.7–6.9) for 36 mm	Age, sex, femoral fixation, femoral head material, year of surgery, and surgical approach
2024	English	Cohort study	1999 – December 2018	New Zealand	Osteoarthritis	Not reported	Not reported	Not reported	Not reported	48,485 (38,989/9,496)	13,190/6,137	25,799/3,359	Revision for all causes	Mean 4.43 for 32 mm and mean 3.70 for 36 mm	Age, sex, approach, implant fixation, and type of polyethylene
2018	Tsikandylakis	Cohort study	2003–2014	Denmark, Finland, Sweden, and Norway*	Osteoarthritis	Mean 70.1 (SD^ 10) for 32 mm and mean 68.9 (SD 10) for 36 mm	39:61/52:48	Not reported	Not reported	85,137 (57,853/27,284)	0/0	All procedures performed involved MoP ~ bearing	Revision for all causes and revision for dislocation	Median 2.8 years (range 0–12) for 32 mm and 2.1 years (range 0–12) for 36 mm	Age, sex, year of procedure, approach, cup and stem fixation, and type of polyethylene
2020	Tsikandylakis	Cohort study	January 1995 – December 2016	Denmark, Finland, Sweden, and Norway*	Proximal femur fracture	Mean 70 ± 11/71 ± 11	38:62/42:58	Not reported	Not reported	5,030 (2,515/2,515)	407/363	2,108/2,152	Revision for all causes and revision for dislocation	Median 2.4 years (interquartile range 0.9–4.4) and 2.6 years (interquartile range 1.1–4.3)	Age, sex, year of procedure, type of fixation, type of bearing, and surgical approach
2023	National Joint Registry (NJR)	Registry report	April 2003 – December 2022	England, Wales, and Northern Ireland	≥ 91.52% osteoarthritis*	Not reported	Not reported	Not reported	Not reported	652,996 (440,468/212,528)	163,627/107,653	276,841/104,875	Revision for all causes computed via data reconstruction	Median 4.69 (IQR 2.17–7.44) for 32 mm and median 4.22 (IQR 1.81–7.05) for 36 mm	None
2023	Swiss National Hip & Knee Joint Registry	Registry report	2012–2022	Switzerland	Osteoarthritis	Not reported	Not reported	Not reported	Not reported	Not reported	Not reported	Not reported	Revision for all causes computed via data reconstruction	Up to 10 years	None

### Study characteristics and study quality

All included studies were observational and analysed arthroplasty registry data from Australia, Denmark, Sweden, Norway, Finland, England, Wales, Northern Ireland, and New Zealand. Table 2 details the results of the bias assessment; no exclusion of any of the eligible studies was required. Where reported or deducible, the mean or median baseline age of participants ranged from 68.9 to 71 years, while the mean or median follow-up duration varied from 2.1 to 4.7 years, with interquartile range values between 0.9 and 7.7 years. Of note, the Swiss National Hip & Knee Joint Registry Report presented results at 10 years post-operatively [44]. Four publications reported data on patients who underwent THR for osteoarthritis only [14, 40, 42, 44]. One article reported on results following THR for proximal femoral fracture [43], while another included data on procedures performed for any indication with the majority being osteoarthritis (approximately ≥ 91.52%) [21]. Two studies exclusively analysed procedures performed using cross-linked polyethylene liners only [40, 43], while another two studies statistically adjusted their results by type of polyethylene liner used (conventional or cross-linked) [41, 42]. The other two articles did not report on this information [21, 44].

**Table 2:** Summary of judgements about each risk of bias category for each included study

First author (Year)	Selection	Comparability	Outcome	Total Score
Hoskins (2022)	★★★★	★★	★★★	9
English (2024)	★★★★	★★	★★★	9
Tsikandylakis (2018)	★★★★	★★	★★★	9
Tsikandylakis (2020)	★★★★	★★	★★★	9
National Joint Registry of England, Wales, and Northern Ireland (2023)	★★★★		★★★	7
Swiss National Hip & Knee Joint Registry (2023)	★★★★		★★★	7

### Revision for dislocation

**Fig. 2:**
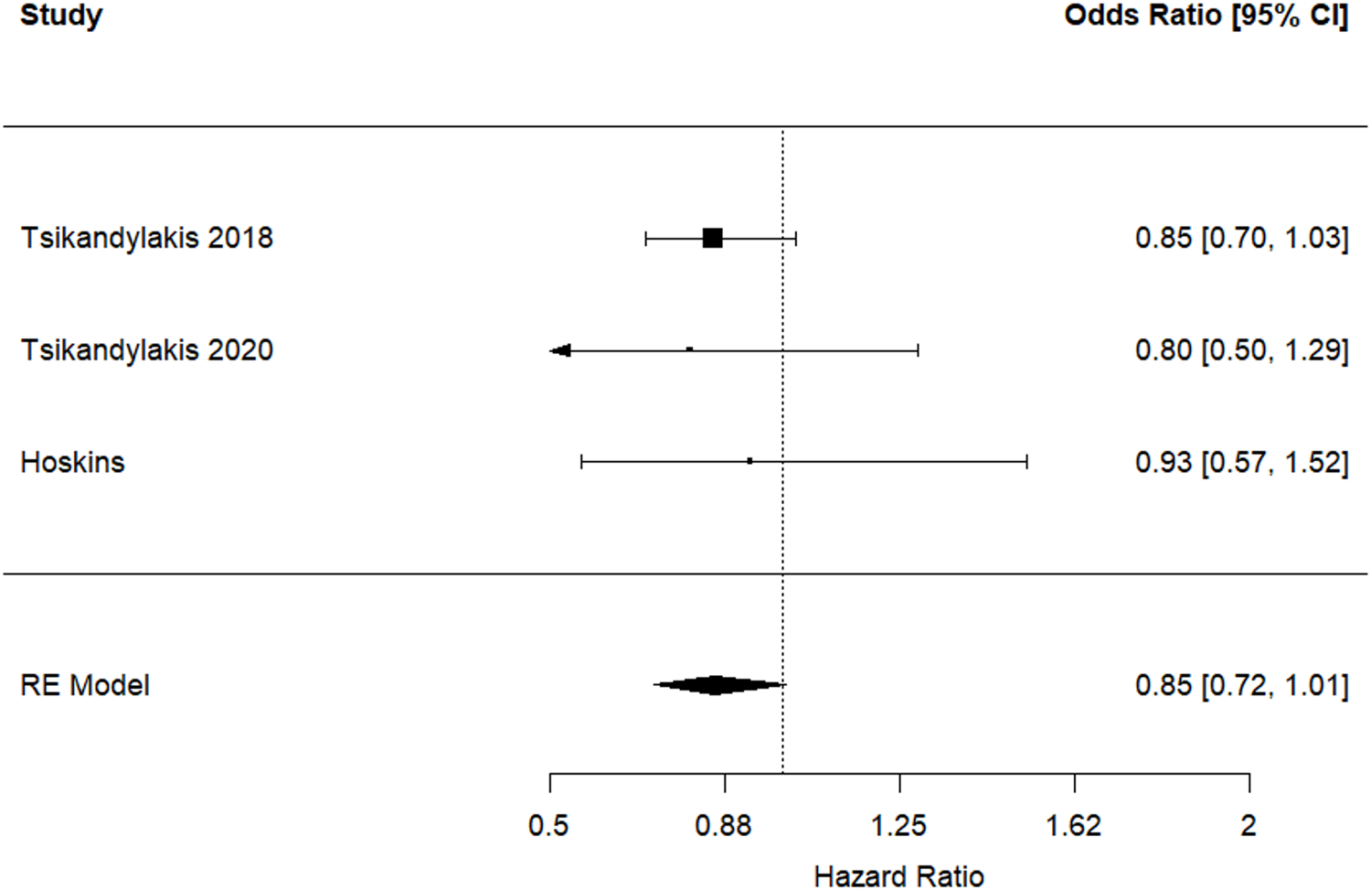
Hazard ratios and 95% confidence interval for the comparison of revision for dislocation following THR performed using 36-mm versus 32-mm femoral head

Pooled analysis demonstrated a marginal statistically significant reduction in risk of revision for dislocation favouring 36-mm over 32-mm femoral heads (HR 0.85, 95% CI 0.72 to 1.01, *p* = 0.058) (Fig. 2). This analysis included 144,759 and 96,377 THR procedures performed using 32-mm and 36 mm femoral heads respectively. There was no significant evidence of between-study heterogeneity (I^2^ = 0%; *p* = 0.911).

### Revision for dislocation (sensitivity analysis)

**Fig. 3:**
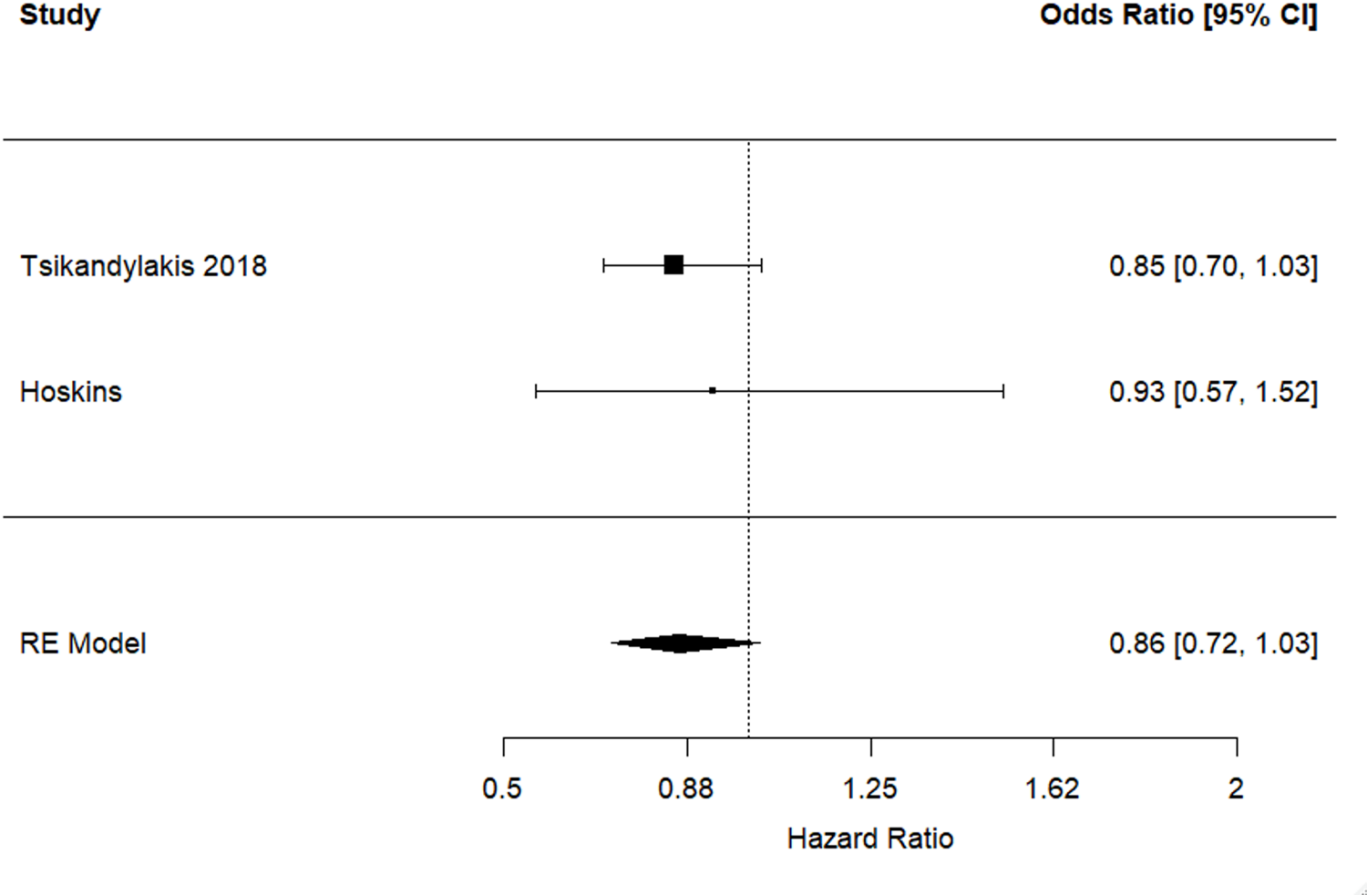
Hazard ratios and 95% confidence intervals for the comparison of revision for dislocation following THR performed for osteoarthritis using 36-mm versus 32-mm femoral heads, and cross-linked polyethylene liners or statistically adjustment for this factor

Similar results were found in the sensitivity analysis for revision due to dislocation, which was limited to THR performed for osteoarthritis using cross-linked polyethylene liners or statistically adjusting for this factor. The hazard ratio for this pooled analysis was 0.86 (95% CI 0.72 to 1.03, *p* = 0.092) (Fig. 3). There were 142,244 and 93,862 THR procedures performed using 32-mm and 36-mm femoral heads respectively that were included in this analysis. There was no significant evidence of between-study heterogeneity (I^2^ = 0%; *p* = 0.740).

### Revision for all-causes

**Fig. 4:**
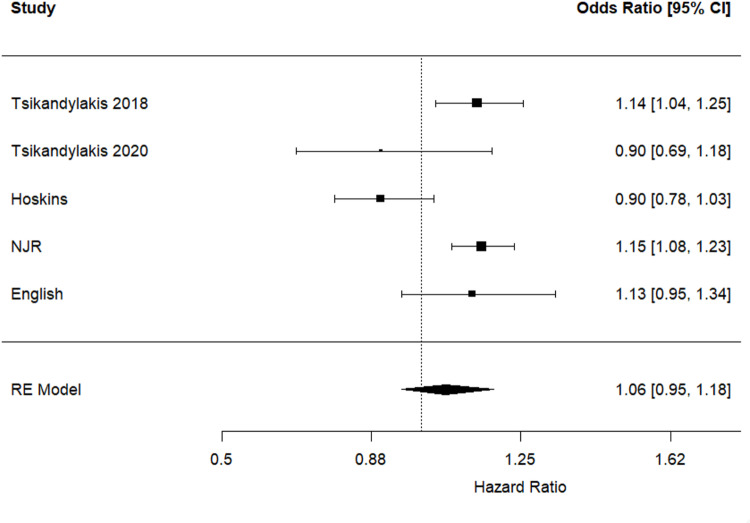
Hazard ratios and 95% confidence interval for the comparison of revision for all-causes following THR performed using 36-mm versus 32-mm femoral head

Pooled analysis showed no statistically significant differences in the risk of revision for all-causes between patients who underwent THR with 36-mm compared to 32-mm femoral heads (HR 1.06, 95% CI 0.95 to 1.18, *p* = 0.287) (Fig. 4). This analysis included 624,216 THR procedures performed using 32-mm femoral heads and 318,401 procedures performed using 36-mm femoral heads. There was considerable statistically significant heterogeneity between the included studies (I^2^ = 73%; *p* = 0.012).

The Swiss National Hip & Knee Joint Registry Report presented the rates of revision for all-causes for uncemented THR performed for osteoarthritis at 10 years post-operatively. Revision rates for ceramic-on-polyethylene bearings were 5.8% (95% CI 4.7 to 7.2) and 4.1% (95% CI 3.4 to 4.9) for 32-mm and 36-mm femoral heads respectively.

### Revision for all-causes (sensitivity analysis)

**Fig. 5:**
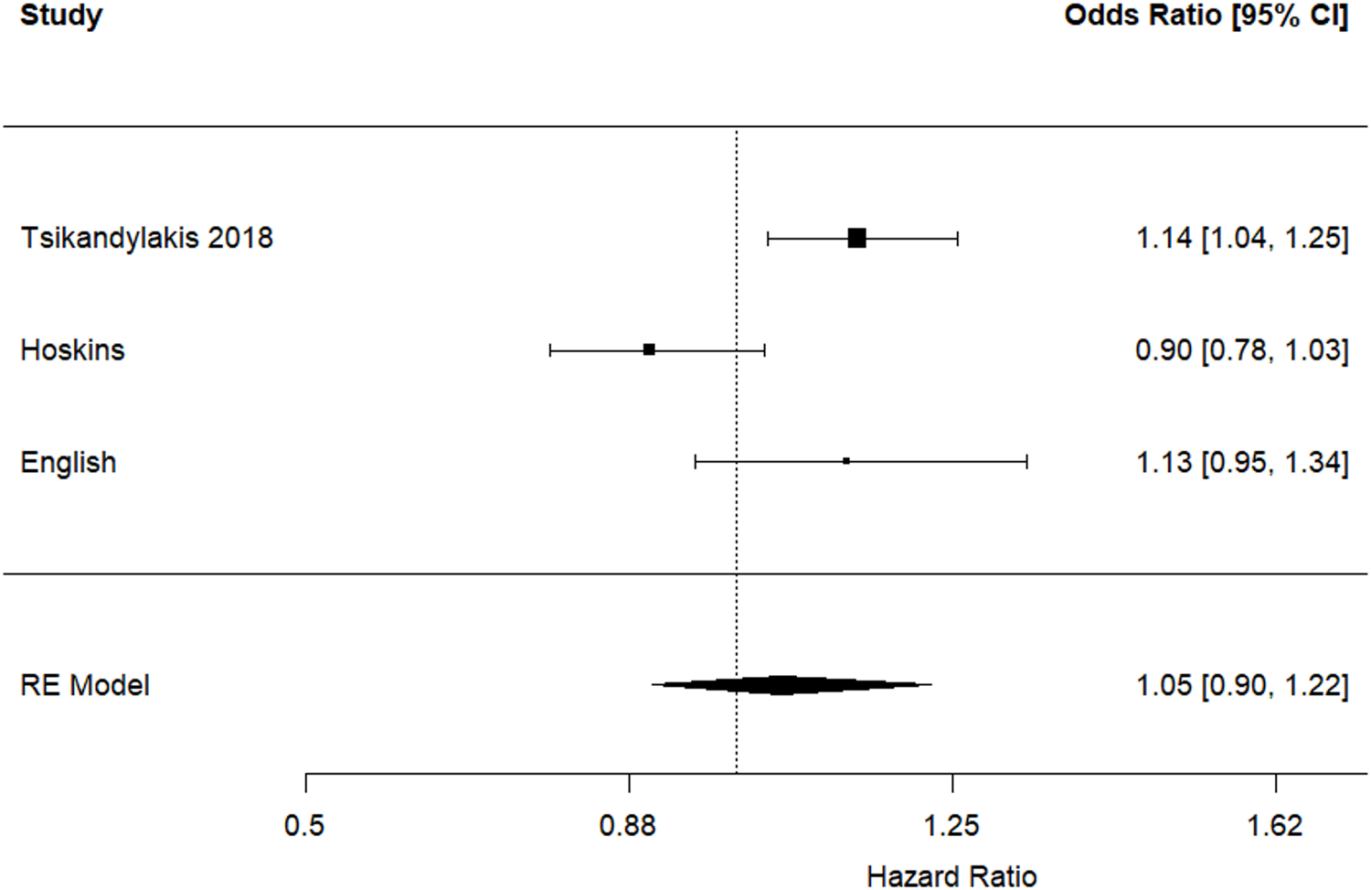
Hazard ratios and 95% confidence intervals for the comparison of revision for all-causes following THR performed for osteoarthritis using 36-mm versus 32-mm femoral heads, and cross-linked polyethylene liners or statistically adjustment for this factor

Restricting the analysis to THR procedures performed for osteoarthritis and using cross-linked polyethylene liners, or statistically adjusting for this factor, also showed no statistically significant differences in the risk of revision for all causes between 36-mm and 32-mm femoral heads (hazard ratio 1.05, 95% CI 0.90 to 1.22, *p* = 0.527) (Fig. 5). Pooled analysis enabled inclusion of 181,233 and 103,358 performed using 32-mm and 36-mm THR respectively. There was considerable statistically significant heterogeneity between the included studies (I^2^ = 75%; *p* = 0.017).

The Swiss National Hip & Knee Joint Registry Report also presented 10-year all-cause revision rates for uncemented THR performed using cross-linked polyethylene liners in patients with osteoarthritis. For ceramic-on-polyethylene bearings, revision rates were 4.2% (95% CI 3.9 to 4.5) for 32-mm and 4.0% (95% CI 3.5 to 4.4) for 36-mm femoral heads. For metal-on-polyethylene bearings, revision rates were 5.3% (95% CI 4.8 to 6.0) for 32-mm and 4.3% (95% CI 3.5 to 5.3) for 36-mm heads.

## Discussion

This is the first systematic review and meta-analysis to evaluate outcomes revision for dislocation and all-causes following THR, comparing 36-mm and 32-mm femoral heads. The findings of the main analysis of early to midterm follow-up data demonstrated a marginal statistically significant reduction in revisions due to dislocation in favour of patients who received 36-mm femoral heads. Furthermore, there were no statistically significant differences in revision for all-causes between the patient groups. Similar findings were noted in the sensitivity analyses that limited procedures to those performed for osteoarthritis and involving cross-linked polyethylene liners or statistically adjusting for this factor.

Previous clinical and laboratory studies on this topic have found conflicting results. Matar et al. conducted a retrospective study in patients who underwent THR using various bearing surfaces including ceramic-on-ceramic. Similar to our study’s results, they also found a marginal statistically significant reduction in revision for dislocation when comparing 32-mm (*n* = 4,858) and 36-mm (*n* = 1,951) femoral head sizes at a mean follow-up of approximately five years post-operatively (*p* = 0.09). There was also no differences in revision for all-causes between the patient groups (*p* = 0.629) [45]. In contrast, van Steenbergen et al. analysed THRs within the Dutch Arthroplasty Register, where most procedures were performed using ceramic-on-polyethylene and metal-on-polyethylene bearings. Their study found that while 36-mm femoral heads (*n* = 50,982) were associated with a lower risk of revision for dislocation compared to 32-mm heads (*n* = 134,910) (HR 0.68, 95% CI 0.59 to 0.78), they had a relatively higher risk of revision for other causes (HR 1.14, 95% CI 1.07 to 1.23) [46]. Alternative study designs such as laboratory studies including finite element analysis have found increased trunnion stresses as femoral head sizes increase from 28-mm to 36-mm diameters [47, 48]. However, a retrieval analysis study of 154 metal-on-polyethylene implants found no association between head size (ranging from 22 mm to 44 mm) and fretting or corrosion damage, as assessed by two independent observers [49]. Lindalen et al. utilised markerless radiostereometric analysis to evaluate wear in patients who were randomised to receive cementless THR with either a 32-mm or 36-mm ceramic head in combination with Vitamin E infused HXLPE liner. At six years post-operatively, they found that the total three-dimensional wear was significantly lower among patients who received 36-mm femoral heads (mean 0.184 mm ± 0.086 SD versus mean 0.290 mm ± 0.170 SD, *p* = 0.015). However, when adjusting their results to account for a three-month period of ‘bedding-in’, these differences were no longer statistically significant (mean 0.023 mm ± 0.114 SD versus mean 0.090 mm ± 0.229 SD, *p* = 0.12) [50].

There are several strengths to this study including the large number of procedures that were analysed, which allowed for the detection of differences in rare events such as revision surgery between patient groups. The inclusion of procedures performed by various surgeons across multiple countries enhanced the study’s external validity, while the analyses of registry datasets provided valuable insights at the population level. Furthermore, by analysing both outcomes revision for dislocation and all-causes, this study provided an understanding of both the potential benefits and risks of using 32-mm compared to 36-mm femoral head sizes. In contrast to other studies, which often grouped a range of head sizes together, this study directly compared two specific head sizes, thereby reducing heterogeneity and providing results that are more directly applicable to clinical practice. The conducted sensitivity analyses accounted for several important covariates, such as the type of polyethylene and surgical approach, thus ensuring a robust assessment of the results.

There are also limitations to this study. Owing to the observational design of the included studies, the results may have been influenced by confounding by indication and unaccounted confounders, particularly in the main analyses. Although the studies included in the sensitivity analyses adjusted for multiple covariates, the specific covariates varied slightly between them. Also, these adjustments did not account for factors such as the use of lipped and highly irradiated polyethylene liners or differences in liner thickness [51, 52]. While randomised controlled trials are effective in addressing confounding, they may not be feasible for this research question given that revision surgery is a rare event. Detecting meaningful differences would require analysing many procedures, making such trials impractical. Although this study combined the results for metal and ceramic femoral heads, neither the subanalyses by bearing type within the included studies nor previous randomised controlled trials have shown these impact revision rates [40, 41, 53]. There is a paucity of studies on this topic and the median/mean follow up for procedures in most of the included studies was less than five years. This duration may be insufficient for detecting differences in revision for all-causes, as certain causes such as aseptic loosening typically occur beyond this timeframe. It is also important to mention that outcomes for total hip replacement (THR) surgery have improved over time, with a notable decline in revision rates [21]. Given that 32-mm femoral heads were more commonly used in earlier years, this trend could have biased results, especially for the comparison of revision for all-causes in the main analysis, towards showing increased revisions within the 32-mm patient group. However, the lack of a significant difference in this outcome between 32-mm and 36-mm groups may provide reassurance regarding the relatively safety of using 36-mm heads up to the follow up period of the included studies.

## Conclusions

At early to midterm follow-up, the use of 36-mm femoral heads may confer a protective effect against the risk of revision for dislocation compared to 32-mm femoral heads, as suggested by the marginal statistically significant reduction observed. However, there were no significant differences the risk of revision for all-causes between these two head sizes. Based on the findings of this study, surgeons seeking to specifically mitigate the risk of revision for dislocation within approximately five years post-operatively should consider using a 36-mm femoral head instead of a 32-mm head. However, caution is warranted, as it is unknown whether revision risk profiles between for two head sizes will differ at relatively longer time points. This uncertainty underscores the urgent need for additional studies to investigate this topic at extended follow up periods.

## Supplementary Information

Below is the link to the electronic supplementary material.

Supplementary Material 1

## Data Availability

No datasets were generated or analysed during the current study.
