# Supplementary material for: Revision for dislocation and all-causes following primary total hip replacement using 36-mm versus 32-mm femoral heads on polyethylene liners: a systematic review and meta-analysis
Source: Arch Orthop Trauma Surg. 2025 Dec 15;146(1):11. doi: 10.1007/s00402-025-06151-w (PMC12705727; doi:10.1007/s00402-025-06151-w)
Supplement: Supplementary file 1 — Supplementary Material 1 [file 402_2025_6151_MOESM1_ESM.docx]

| Medline and Embase | 1. ARTHROPLASTY, REPLACEMENT, HIP/  2. reoperation/  3. Prosthesis Failure/  4. revis*.ti,ab.  5. (Hip* adj3 (replace* or arthroplast* or prosth* or endoprosth*)).ab. or (Hip* adj3 (replace* or arthroplast* or prosth* or endoprosth*)).ti.  6. 1 or 5  7. head*.ti,ab.  8. 2 or 3 or 4  9. 6 and 7 and 8  10. limit 9 to yr="1995 -Current" |
| --- | --- |
| The Cochrane Library | #1 MeSH descriptor: [Arthroplasty, Replacement, Hip] explode all trees  #2 MeSH descriptor: [Reoperation] explode all trees  #3 MeSH descriptor: [Prosthesis Failure] explode all trees  #4 (revis*):ti,ab  #5 (Hip* near/3 (replace* or arthroplast* or prosth* or endoprosth*)):ti,ab  #6 (head*):ti,ab  #7 #1 or #5  #8 #2 or #3 or #4  #9 #6 and #7 and #8 |
| Web of Science | ((Hip* or knee* or joint*) NEAR/3 (replace* or arthroplast* or prosth* or endoprosth*)) (Topic) and revis* (Topic) and head* (Topic) |

Table S1: Medical Subject Headings and free words used in the literature search across the four databases.
